# Supplementary material for: A mathematical model for predicting glucose levels in critically-ill patients: the PIGnOLI model
Source: PeerJ. 2015 Jun 9;3:e1005. doi: 10.7717/peerj.1005 (PMC4465940; doi:10.7717/peerj.1005)
Supplement: Supplemental Information 1 [file peerj-03-1005-s001.docx]

# Population

select * from icustay_detail

where icustay_age_group like 'adult'

//32332

# Comorbidity

select * from COMORBIDITY_SCORES

# laboratory findings

select *

FROM labevents

INNER JOIN d_labitems

ON labevents.itemid=d_labitems.itemid

where (d_labitems.LOINC_DESCRIPTION like '%Bilirubin%' AND d_labitems.FLUID like 'BLOOD'）

//162849

select *

FROM labevents

INNER JOIN d_labitems

ON labevents.itemid=d_labitems.itemid

where d_labitems.test_name like '%CRP%'

//4358

select *

FROM labevents

INNER JOIN d_labitems

ON labevents.itemid=d_labitems.itemid

where d_labitems.LOINC_DESCRIPTION like '%Creatinine%'AND d_labitems.FLUID like 'BLOOD' AND d_labitems.test_name like '%CREAT%'

//526309

select *

FROM labevents

INNER JOIN d_labitems

ON labevents.itemid=d_labitems.itemid

where d_labitems.TEST_NAME like 'LACTATE'

//104362

# Blood glucose (fingerstick)

select * from d_chartitems

where label like '%glucose%'

1 1455 fingerstick glucose

2 1812 abg: glucose

3 2338 finger stick glucose

select * from d_chartitems

where label like '%Glucose%'

1 807 Fingerstick Glucose Chemistry

2 811 Glucose (70-105) Chemistry

3 3447 Glucose Monitor # (null)

4 3744 Blood Glucose Chemistry

5 3745 BloodGlucose Quick Admit

6 3816 Urine Glucose Urine

7 1529 Glucose Chemistry

select * from chartevents

where itemid=1812 OR itemid=1812 OR itemid=1455 OR itemid=2338 OR itemid=807 OR itemid=811 OR itemid=3744 OR itemid=3745 OR itemid=1529

SELECT *

FROM chartevents

INNER JOIN d_chartitems

ON chartevents.itemid=d_chartitems.itemid

where d_chartitems.label like '%glucose%' OR (d_chartitems.label like '%Glucose%' AND (d_chartitems.category like '%Chemistry%' OR d_chartitems.category like 'Quick Admit'))

//1117076

# Insulin therapy

select * from d_meditems

where label like '%Insulin%'

| 1 | 100 | Regular Insulin |
| --- | --- | --- |
| 2 | 310 | Insulin Drip |
| 3 | 45 | Insulin |

select * from medevents

where itemid=310 OR itemid=45

//480560

select * from d_ioitems

where label like '%insulin%'

| 1 | 280 | insulin pump u/hr | Free Form Intake |
| --- | --- | --- | --- |
| 2 | 4354 | insulin carrier | Free Form Intake |

select * from d_ioitems

where label like '%Insulin%'

| 1 | 209 | Insulin U/hr | IV Drips |
| --- | --- | --- | --- |
| 2 | 211 | .9% Normal Saline 100.0ml + 100Uhr Insulin | IV Infusions |
| 3 | 231 | .9% Normal Saline 50.0ml + 50Uhr Insulin | IV Infusions |
| 4 | 271 | .9% Normal Saline 50.0ml + 100Uhr Insulin | IV Infusions |
| 5 | 419 | .9% Normal Saline 100.0ml + 50Uhr Insulin | IV Infusions |
| 6 | 466 | .9% Normal Saline 250.0ml + 250Uhr Insulin | IV Infusions |
| 7 | 709 | D5W 100.0ml + 50Uhr Insulin | IV Drips |
| 8 | 829 | .9% Normal Saline 500.0ml + 500Uhr Insulin | IV Infusions |
| 9 | 3550 | D10W 50.0cc + 5Ukghr Insulin Drip | IV Drips |
| 10 | 3551 | Insulin | IV Drips |
| 11 | 871 | .9% Normal Saline 100.0ml + 150Uhr Insulin | IV Infusions |
| 12 | 882 | .9% Normal Saline 500.0ml + 100Uhr Insulin | IV Infusions |
| 13 | 1286 | .9% Normal Saline 100.0ml + 25Uhr Insulin | IV Drips |
| 14 | 4518 | Insulin Carrier | Free Form Intake |
| 15 | 2843 | .9% Normal Saline 250.0ml + 500Uhr Insulin | IV Infusions |
| 16 | 2860 | .9% Normal Saline 200.0ml + 100Uhr Insulin | IV Infusions |
| 17 | 3146 | .9% Normal Saline 200.0ml + 200Uhr Insulin | IV Infusions |
| 18 | 4234 | D5W 100.0ml + 200Uhr Insulin | IV Drips |
| 19 | 5186 | Insulin carrier | Free Form Intake |
| 20 | 5322 | NS Insulin Carrier | Free Form Intake |
| 21 | 6719 | .9% Normal Saline 50.0ml + 500Uhr Insulin | IV Infusions |
| 22 | 747 | .9% Normal Saline 500.0ml + 50Uhr Insulin | IV Infusions |
| 23 | 1060 | .9% Normal Saline 50.0ml + 25Uhr Insulin | IV Drips |
| 24 | 1300 | D5W 100.0ml + 100Uhr Insulin | IV Infusions |
| 25 | 447 | .9% Normal Saline 100.0ml + 200Uhr Insulin | IV Drips |

select * from ioevents

where itemid=280 OR itemid=4354 OR itemid=209 OR itemid=211 OR itemid=231 OR itemid=271 OR itemid=419 OR itemid=466 OR itemid=709 OR itemid=829 OR itemid=3550 OR itemid=3551 OR itemid=871 OR itemid= 882 OR itemid=1286 OR itemid=4518 OR itemid=2843 OR itemid=2860 OR itemid=3146 OR itemid=4234 OR itemid=5186 OR itemid=5322 OR itemid=6719 OR itemid=747 OR itemid=1060 OR itemid=1300 OR itemid=447

select * from deliveries

where ioitemid=280 OR ioitemid=4354 OR ioitemid=209 OR ioitemid=211 OR ioitemid=231 OR ioitemid=271 OR ioitemid=419 OR ioitemid=466 OR ioitemid=709 OR ioitemid=829 OR ioitemid=3550 OR ioitemid=3551 OR ioitemid=871 OR ioitemid= 882 OR ioitemid=1286 OR ioitemid=4518 OR ioitemid=2843 OR ioitemid=2860 OR ioitemid=3146 OR ioitemid=4234 OR ioitemid=5186 OR ioitemid=5322 OR ioitemid=6719 OR ioitemid=747 OR ioitemid=1060 OR ioitemid=1300 OR ioitemid=447

# IV dextrose

SELECT *

FROM deliveries

INNER JOIN d_ioitems

ON deliveries.ioitemid=d_ioitems.itemid

where (d_ioitems.label like '%D5W%' OR d_ioitems.label like '%Dextrose%' OR d_ioitems.label like '%dextrose%'OR d_ioitems.label like '%D10W%' OR d_ioitems.label like '%D20W%') AND d_ioitems.category like '%IV%'

SELECT *

FROM IOEVENTS

INNER JOIN d_ioitems

ON IOevents.itemid=d_ioitems.itemid

where d_ioitems.label like '%TPN%'

//579518

# PO feeding

select *

FROM deliveries

INNER JOIN d_ioitems

ON deliveries.ioitemid=d_ioitems.itemid

where (d_ioitems.label like '%D5W%' OR d_ioitems.label like '%Dextrose%' OR d_ioitems.label like '%dextrose%'OR d_ioitems.label like '%D10W%' OR d_ioitems.label like '%D20W%') AND d_ioitems.category like '%PO%'

//192 items

select *

FROM deliveries

INNER JOIN d_ioitems

ON deliveries.ioitemid=d_ioitems.itemid

where d_ioitems.category like '%PO%'

// 973 items

select *

FROM deliveries

INNER JOIN d_ioitems

ON deliveries.ioitemid=d_ioitems.itemid

where d_ioitems.label like '%Tube%' OR d_ioitems.label like '%tube%'

//680

select *

FROM deliveries

INNER JOIN d_ioitems

ON deliveries.ioitemid=d_ioitems.itemid

where d_ioitems.label like '%Po Intake%'

//13

select *

FROM ioevents

INNER JOIN d_ioitems

ON ioevents.itemid=d_ioitems.itemid

where d_ioitems.label like '%Tube%' OR d_ioitems.label like '%tube%'

//339328

select *

FROM ioevents

INNER JOIN d_ioitems

ON ioevents.itemid=d_ioitems.itemid

where d_ioitems.label like '%Po Intake%'

//190937

select *

FROM ioevents

INNER JOIN d_ioitems

ON ioevents.itemid=d_ioitems.itemid

where (d_ioitems.label like '%D5W%' OR d_ioitems.label like '%Dextrose%' OR d_ioitems.label like '%dextrose%'OR d_ioitems.label like '%D10W%' OR d_ioitems.label like '%D20W%') AND d_ioitems.category like '%PO%'

//29705

select *

FROM ioevents

INNER JOIN d_ioitems

ON ioevents.itemid=d_ioitems.itemid

where d_ioitems.category like '%PO%'

//803163

Summary of the po intake by different searching strategies

| Method | Ioevents | deliveries |
| --- | --- | --- |
| All “PO”^¶^ | 803163 | 973 |
| Dextroase intake | 29705 | 192 |
| Po intake | 190937 | 13 |
| Tube feeding | 339328 | 680 |

^¶^ Including fluid containing no dextrose such as drugs, normal saline and so on.

select *

FROM ioevents

INNER JOIN d_ioitems

ON ioevents.itemid=d_ioitems.itemid

where ((d_ioitems.label like '%D5W%' OR d_ioitems.label like '%Dextrose%' OR d_ioitems.label like '%dextrose%'OR d_ioitems.label like '%D10W%' OR d_ioitems.label like '%D20W%') AND d_ioitems.category like '%PO%') OR d_ioitems.label like '%Po Intake%' OR d_ioitems.label like '%Tube%'

//558634
