# Supplementary material for: A mathematical model for predicting glucose levels in critically-ill patients: the PIGnOLI model
Source: PeerJ. 2015 Jun 9;3:e1005. doi: 10.7717/peerj.1005 (PMC4465940; doi:10.7717/peerj.1005)
Supplement: Supplemental Information 3 [file peerj-03-1005-s003.docx]

Supplemental Digital Content 2

Figure 1 is the leverage versus residual squared plot. Using residual squared instead of residual itself, the graph is restricted to the first quadrant and the relative positions of data points are preserved. This is a quick way of checking potential influential observations and outliers at the same time (points located to the upper right corner). Cook's D and DFITS were overall measure of influence of a certain observation (figure 2). Figure 2 shows that several points are far away from the remaining, indicating influential points.

#
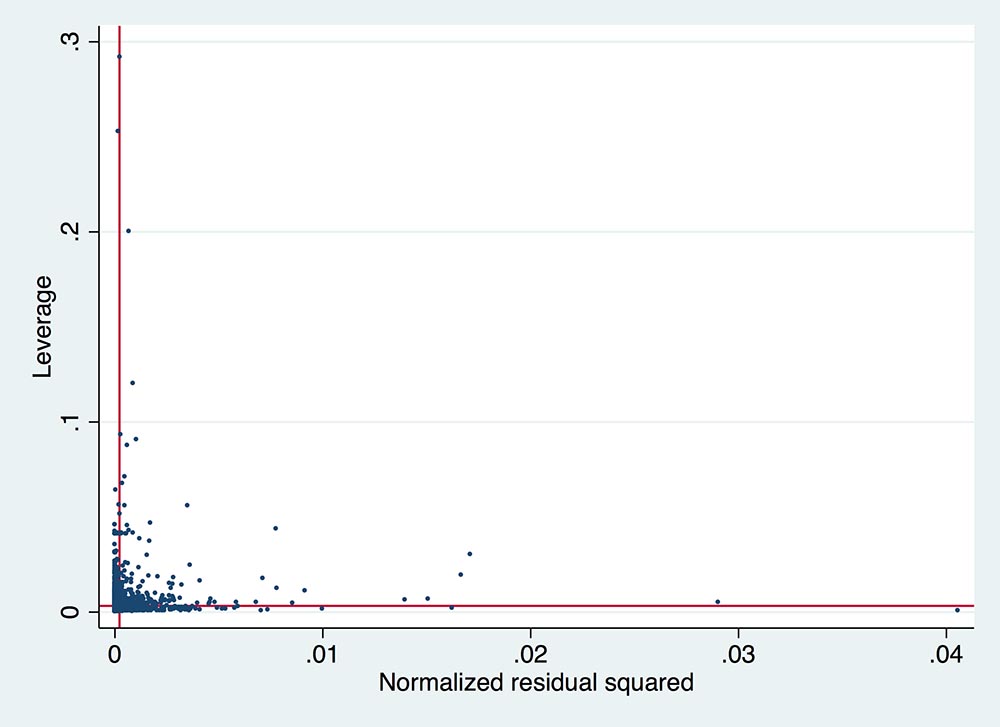


Figure 1. The leverage versus residual squared plot. Using residual squared instead of residual itself, the graph is restricted to the first quadrant and the relative positions of data points are preserved. This is a quick way of checking potential influential observations and outliers at the same time (points located to the upper right corner).


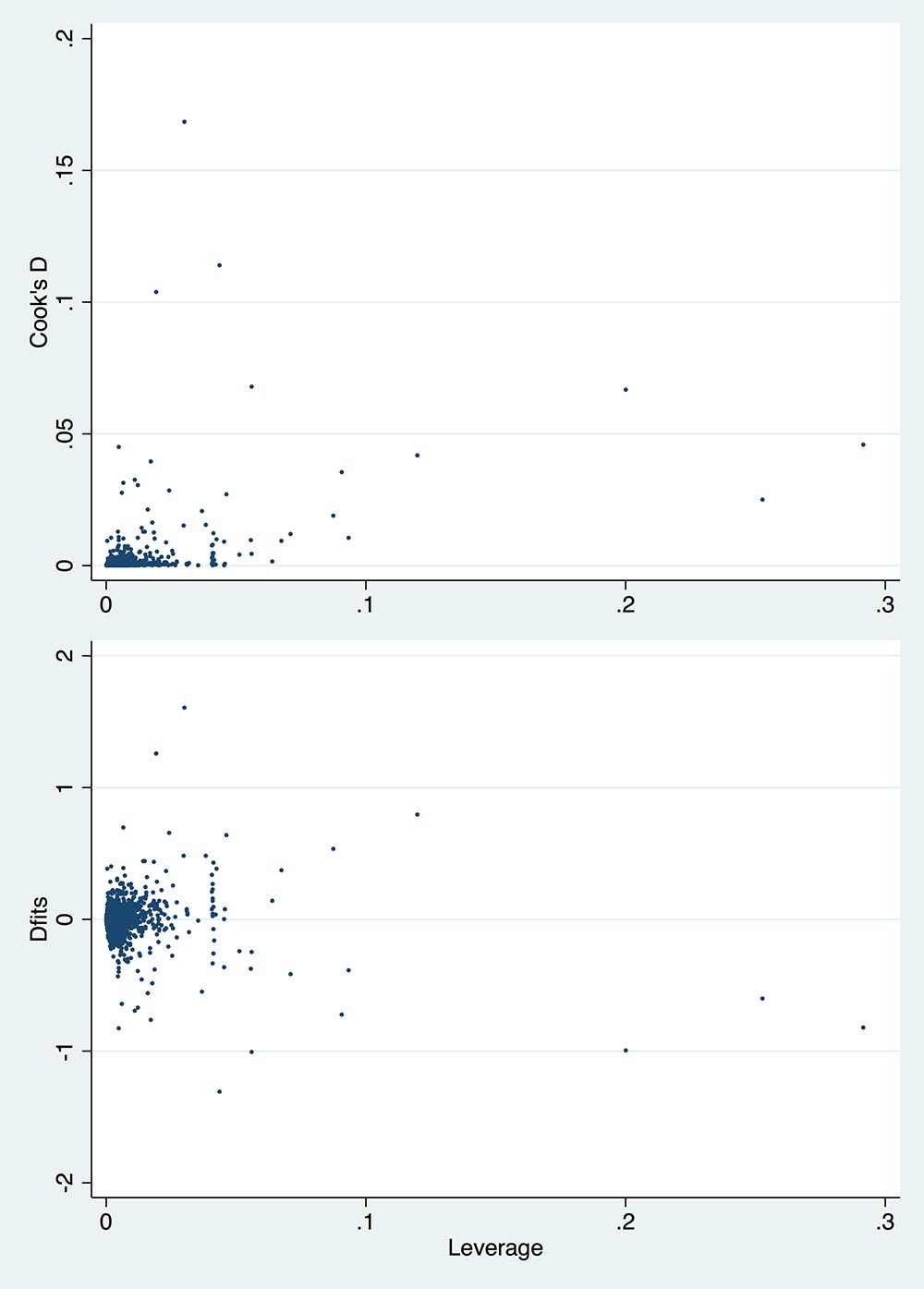


Figure 2. Cook's D and DFITS were overall measure of influence of a certain observation. The figure shows that several points are far away from the remaining, indicating influential points.


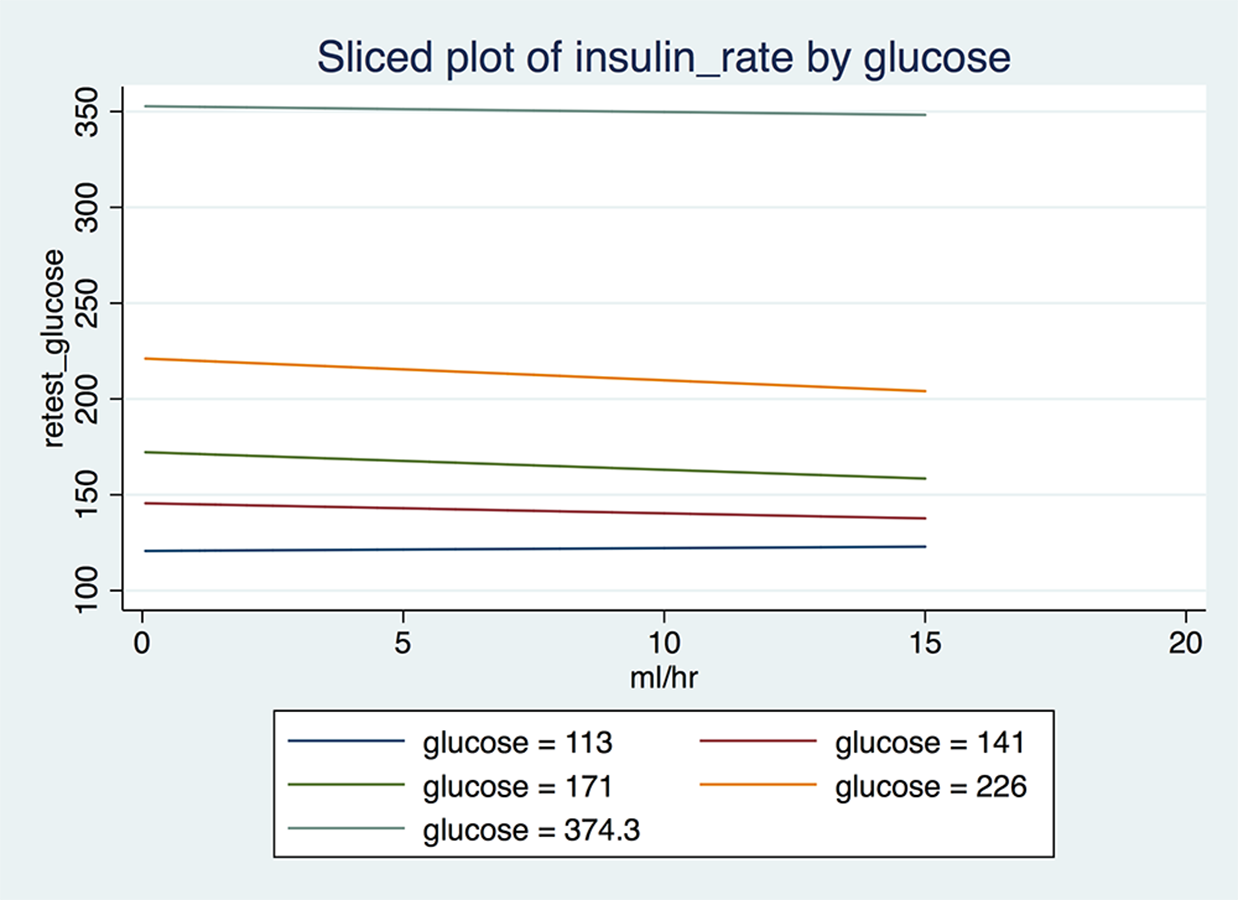


Figure 3. Graphical presentation of interaction between BG and insulin rate. Although this interaction was statistically significant, the magnitude was of marginal clinical significance.
